# Supplementary material for: Modeling glioblastoma heterogeneity as a dynamic network of cell states
Source: Mol Syst Biol. 2021 Sep 16;17(9):e10105. doi: 10.15252/msb.202010105 (PMC8444284; doi:10.15252/msb.202010105)
Supplement: Supplementary file 6 — Source Data for Figure 5 [file MSB-17-e10105-s004.zip › Figure5A_sourcedata/GSEA_3071/nosigdb_stateIII.GseaPreranked.1622795155562/ANASTASSIOU_MULTICANCER_INVASIVENESS_PMID_22208948.html]

Details for gene set ANASTASSIOU\_MULTICANCER\_INVASIVENESS\_PMID\_22208948[GSEA]

|  || Dataset | state33071 |
| Phenotype | NoPhenotypeAvailable |
| Upregulated in class | na\_neg |
| GeneSet | ANASTASSIOU\_MULTICANCER\_INVASIVENESS\_PMID\_22208948 |
| Enrichment Score (ES) | -0.67988634 |
| Normalized Enrichment Score (NES) | -3.3988261 |
| Nominal p-value | 0.0 |
| FDR q-value | 0.0 |
| FWER p-Value | 0.0 |
Table: GSEA Results Summary

  

Fig 1: Enrichment plot: ANASTASSIOU\_MULTICANCER\_INVASIVENESS\_PMID\_22208948      
 Profile of the Running ES Score & Positions of GeneSet Members on the Rank Ordered List

  

| PROBE | GENE SYMBOL | GENE\_TITLE | RANK IN GENE LIST | RANK METRIC SCORE | RUNNING ES | CORE ENRICHMENT || 1 | LGALS1 |  |  | 230 | 0.323 | -0.0460 | No |
| 2 | THY1 |  |  | 472 | -0.253 | -0.0977 | No |
| 3 | NTM |  |  | 553 | -0.257 | -0.1079 | No |
| 4 | PRRX1 |  |  | 1032 | -0.281 | -0.2194 | No |
| 5 | AEBP1 |  |  | 1763 | -0.322 | -0.3939 | No |
| 6 | RCN3 |  |  | 1906 | -0.330 | -0.4170 | No |
| 7 | ITGBL1 |  |  | 2093 | -0.341 | -0.4510 | No |
| 8 | SERPINF1 |  |  | 2245 | -0.352 | -0.4756 | No |
| 9 | MMP2 |  |  | 2527 | -0.375 | -0.5326 | No |
| 10 | ACTA2 |  |  | 2721 | -0.393 | -0.5663 | No |
| 11 | ASPN |  |  | 2821 | -0.401 | -0.5755 | No |
| 12 | NUAK1 |  |  | 2920 | -0.410 | -0.5841 | No |
| 13 | OLFML2B |  |  | 3050 | -0.425 | -0.6000 | No |
| 14 | EDNRA |  |  | 3351 | -0.471 | -0.6581 | Yes |
| 15 | SPOCK1 |  |  | 3372 | -0.474 | -0.6440 | Yes |
| 16 | PLAU |  |  | 3435 | -0.488 | -0.6402 | Yes |
| 17 | MFAP5 |  |  | 3536 | -0.523 | -0.6447 | Yes |
| 18 | COL6A2 |  |  | 3674 | -0.595 | -0.6558 | Yes |
| 19 | PDGFRB |  |  | 3678 | -0.598 | -0.6323 | Yes |
| 20 | DCN |  |  | 3723 | -0.633 | -0.6180 | Yes |
| 21 | COL5A2 |  |  | 3726 | -0.636 | -0.5928 | Yes |
| 22 | VCAN |  |  | 3741 | -0.660 | -0.5696 | Yes |
| 23 | TIMP3 |  |  | 3755 | -0.677 | -0.5456 | Yes |
| 24 | PCOLCE |  |  | 3777 | -0.708 | -0.5223 | Yes |
| 25 | TNFAIP6 |  |  | 3780 | -0.713 | -0.4939 | Yes |
| 26 | CDH11 |  |  | 3789 | -0.721 | -0.4668 | Yes |
| 27 | FBN1 |  |  | 3790 | -0.723 | -0.4375 | Yes |
| 28 | INHBA |  |  | 3801 | -0.746 | -0.4099 | Yes |
| 29 | SPARC |  |  | 3811 | -0.759 | -0.3815 | Yes |
| 30 | BGN |  |  | 3812 | -0.761 | -0.3507 | Yes |
| 31 | COL5A1 |  |  | 3815 | -0.764 | -0.3203 | Yes |
| 32 | COL11A1 |  |  | 3828 | -0.788 | -0.2914 | Yes |
| 33 | FN1 |  |  | 3850 | -0.842 | -0.2627 | Yes |
| 34 | LUM |  |  | 3861 | -0.870 | -0.2301 | Yes |
| 35 | POSTN |  |  | 3885 | -1.025 | -0.1945 | Yes |
| 36 | COL6A3 |  |  | 3900 | -1.171 | -0.1507 | Yes |
| 37 | COL1A1 |  |  | 3902 | -1.191 | -0.1027 | Yes |
| 38 | COL1A2 |  |  | 3906 | -1.278 | -0.0518 | Yes |
| 39 | COL3A1 |  |  | 3918 | -1.418 | 0.0028 | Yes |
Table: GSEA details [plain text format]

  

Fig 2: ANASTASSIOU\_MULTICANCER\_INVASIVENESS\_PMID\_22208948: Random ES distribution      
 Gene set null distribution of ES for **ANASTASSIOU\_MULTICANCER\_INVASIVENESS\_PMID\_22208948**
